# Editorial: Malaria molecular epidemiology current situation in Africa

**DOI:** 10.3389/fepid.2024.1400612

**Published:** 2024-04-03

**Authors:** Kwesi Zandoh Tandoh, Nancy Odurowah Duah-Quashie, Jaishree Raman, Lynette Isabella Ochola-Oyier

**Affiliations:** ^1^ Department of Epidemiology, Noguchi Memorial Institute for Medical Research, University of Ghana, Accra, Ghana; ^2^ Laboratory for Antimalarial Resistance Monitoring and Malaria Operational Research, National Institute of Communicable Diseases (NCID), Johannesburg, South Africa; ^3^ Biosciences Department, Kenya Medical Research Institute (KEMRI) Wellcome Trust Research Programme, Kilifi, Kenya

**Keywords:** *hrp2/3*, drug resistance, molecular epidemiology, molecular surveillance, molecular markers

**Editorial on the Research Topic** Malaria molecular epidemiology current situation in Africa

Malaria remains a significant public health burden in many of the 85 malaria-endemic countries, the majority of which are in sub-Saharan Africa (sSA) (1). The value of molecular surveillance in evidence-based decision-making was clearly demonstrated during the COVID-19 pandemic. National Malaria Control Programmes (NMCPs) across Africa are now using of malaria molecular surveillance and epidemiology data to guide intervention selection and targeting to help them eliminate malaria. The four articles in this research topic highlight the importance of molecular epidemiology in informing and improving malaria surveillance strategies.

One of the threats to malaria elimination is the emergence and spread of *Plasmodium falciparum* parasites with deletions in the histidine-rich protein 2 and 3 (*hrp2/3*) genes. Malaria parasites with these deletions evade detection by *hrp2*-based RDTs, the preferred point-of-care diagnostic across most of sSA, increasing the risk malaria-related morbidity and mortality as well as the chances of onward transmission (2, 3). For evidence-based decision on malaria diagnostics, it is essential that NMCPs have accurate and current data on the prevalence and distribution of parasites carrying these deletions.

Duah-Quashie et al., determined the prevalence of parasites with *hrp2/3* gene deletions in symptomatic children from 10 sentinel sites located across three different ecological regions in Ghana between 2015 and 2020. Sequence data generated from archived dried blood spots were analyzed for deletions and polymorphisms in the *hrp2* and *hrp3* genes. Of the 2,540 samples analyzed, 30.7% carried *hrp2* deletions and 17.2% *hrp3* deletions, with the prevalence of these gene deletions increasing over time. These findings suggested a possible decrease in sensitivity in the ability of *hrp2*-based RDTs to detect malaria in Ghana and calls for increased surveillance.

Similar to Duah-Quashie et al., Okanda et al., investigated the prevalence of *hrp2/3* gene deletions in malaria parasites collected from symptomatic and uncomplicated malaria patients in Kilifi, Kenya between November 2019 and February 2020. From the 345 samples collected none of the 11 RDT-negative and microscopy positive samples carried both the *hrp2* or *hrp3* gene deletion. However, an extension of the criteria to increase the sample size using qPCR positive samples identified a low prevalence of both *hrp2* and *hrp3* gene deleted parasites at 2.1%. The findings from these studies underscore the importance of constant surveillance and the need for novel cost-effective point-of care malaria diagnostic.

The third article in this collection by Matrevi et al., investigated the almost inevitable problem of the emergence and spread of drug-resistant malaria parasites. Artemisinin-based combination therapies (ACTs) are the recommended treatment for uncomplicated malaria as they are fast acting and highly efficacious. Currently these drugs are the most widely used antimalarials in Africa, so the emergence and spread of resistance to ACTs poses a significant risk to sSA's malaria control/elimination efforts (4). Monitoring the prevalence of molecular markers associated with antimalarial resistance enables the early detection of and response to emerging resistance. In their study, Matrevi et al., determined the prevalence of mutations in nine *P. falciparum* genes associated with resistance to artemisinin derivatives, lumefantrine, chloroquine, quinine, sulphadoxine and pyrimethamine in Ghana. The 1,170 parasite samples assessed were collected over five transmission seasons between 2007 and 2018 from symptomatic children aged 9 years and younger with uncomplicated malaria. The prevalence of parasites carrying mutations in the *P. falciparum falcipain 2* gene, potentially associated with artemisinin-partial resistance, increased over the study duration, while no known mutations associated with artemisinin-partial resistance were detected in the *P. falciparum coronin* gene. Mutation in the *P. falciparum cycteine desulfurase* gene, possibly associated with lumefantrine resistance, also increased over the study period. These increases in mutation prevalence may be associated with recent reports of decreasing ACT efficacy in Ghana and highlights the need for sustained molecular surveillance to mitigate the risk of drug resistant parasites becoming established in Ghana.

The final paper of this research topic by Arambepola et al., investigated the impacts of how and when sampling is conducted on the determinants of *P. falciparum* population structure. Malaria genomic data have been used to understand changes in transmission intensity and parasite relatedness. The more closely related parasites are, the closer they are on a transmission network, potentially suggesting a foci of local transmission. This information can be used by NMCPs to inform control strategies. However, in areas of moderate to high transmission, the complexity of infections makes inferring relatedness challenging.

Arambepola et al., used two measures of relatedness to investigate population structure in a moderate transmission setting in Kenya. The model developed was then used to assess the power of genomic data to determine population structure under different sampling schemes, levels of missing data and transmission settings. The study revealed that infections sampled closer in time were more likely to genetically similar and less differentiated compared to those sampled further apart. However, there was limited evidence of spatial (village-level) structure. Power to estimate relatedness decreased as the level of missing data increased but was not impacted when only sampling symptomatic individuals. Data from this study suggest that active dense sampling can detect population structure, even when certain data are missing, but not when there are high levels of connectivity between different regions. More research is required to address these short comings.

Considering other epidemiological factors that were not discussed in the articles under this topic is the invasion of *Anopheles stephensi* into the Horn of Africa which is rapidly spreading in the region into East Africa and as far west into Nigeria and Ghana. This new occurrence calls for effective entomological surveys in African countries as this vector will enhance urban malaria spread and *P. vivax* transmission. It is worth mentioning the promising gains that can be made by the recent exciting efficacy data from the R21 vaccine bringing hope to reduction of malaria prevalence in Africa.

The work presented in this supplement, highlights the value of malaria molecular epidemiology in guiding evidence-based strategic planning and surveillance strategy implementation by NMCPs to advance elimination efforts. Malaria molecular epidemiology is an essential tool in the elimination toolbox of all NMCPs.
